# Mono-symptomatic Fabry disease in a population with mild-to-moderate left ventricular hypertrophy

**DOI:** 10.1016/j.ymgmr.2020.100697

**Published:** 2020-12-10

**Authors:** Maria Fuller, Rebecca Perry, Madiha Saiedi, Janice M. Fletcher, Joseph B. Selvanayagam

**Affiliations:** a Genetics and Molecular Pathology, SA Pathology at Women's and Children's Hospital, 72 King William Road, North Adelaide, South Australia 5006, Australia; b Adelaide Medical School, University of Adelaide, Adelaide, South Australia 5005, Australia; c College of Medicine, Flinders University of South Australia, Australia; d Department of Cardiovascular Medicine, Flinders Medical Centre, Southern Adelaide Local Health Network, Bedford Park, South Australia 5042, Australia; e Cardiac Imaging Research Group, South Australian Health and Medical Research Institute, Adelaide, South Australia 5000, Australia

**Keywords:** Fabry disease, Lysosomal storage disorder, Screening, Left ventricular hypertrophy

## Abstract

Fabry disease (FD) results from a deficiency in the exoglycohydrolase, α-galactosidase A (AGA), an enzyme required for the sequential degradation of glycosphingolipids, which consequently accumulate in the lysosomes of affected cells. An X-linked inherited metabolic disorder, FD has a high incidence of a later onset phenotype that is under-diagnosed and under-recognised in adulthood despite the availability of specific treatment. As the first presenting feature in adults is often left ventricular hypertrophy (LVH), we hypothesized that testing patients with an attenuated echocardiographic phenotype of unexplained hypertrophic cardiomyopathy, might identify cases of undiagnosed FD. We employed a simple screening test by measuring AGA activity in dried blood spots collected from a finger-prick of blood in a cohort of 511 individuals aged between 18 and 75 with LVH between 1.2 and 1.5 cm. Two males were identified with AGA activity below the reference interval and subsequent molecular testing confirmed the commonly reported genetic variants, p.Ala143Thr in one individual and p.Asn215Ser, in the other. Additional biochemical measurement of plasma, lyso-Gb1 was normal in both patients. Of the 179 females screened, one individual returned AGA activity slightly below the reference interval but was lost to further follow-up. This pilot study suggests that screening patients with mild-to-moderate LVH of unknown aetiology does indeed identify undiagnosed cases of FD.

## 1 Introduction

Fabry disease (FD) is an X-linked lysosomal disorder arising from molecular variants in the *GLA* gene producing inadequate α-galactosidase A (AGA) activity. Consequently, the enzyme's substrate, globotriaosylceramide, accumulates predominantly in the vascular endothelium of the heart, kidney, skin and brain. The ‘classical’ phenotype manifests in childhood or early adolescence invariably as acroparesthesiae, angiokeratoma, hypo-anhidrosis and progresses to renal insufficiency, cardiac disease and cerebrovascular events. Later-onset or ‘non-classical’ FD lack these early manifestations, typically exhibiting almost exclusively renal, cardiac or cerebrovascular episodes and therefore these patients are considered mono-symptomatic upon initial presentation [1].

Since initial newborn screening data from both Italy and Taiwan predicted a high incidence of late-onset FD [2,3] a number of screening programs have been conducted targeting patients with an echocardiographic phenotype of left ventricular hypertrophic (LVH) cardiomyopathy on the premise that undiagnosed FD exists within that population. The reanalysis of all cardiac reported screening studies (including both hypertrophic cardiomyopathy (HCM) and LVH) for FD from 1995 through to September 2017 reported 0.9% among both male and female patients. Notably, of these reports, six studies selected patients with only LVH [4]. More recently, 2.5% of patients from a small cohort with unexplained late gadolinium enhancement (LGE) on cardiovascular magnetic resonance imaging (CMR) were found to have FD [5]. Against this background, we hypothesized that testing patients with an attenuated echocardiographic phenotype of unexplained LVH may identify cases of undiagnosed, mono-symptomatic FD. Unlike other studies, we did not exclude patients with coronary artery disease as this can be seen in patients with FD.

## 2 Methods

A total of 4099 participants (2787 men and 1312 women) were selected from an echocardiography database of a major teaching hospital (Flinders Medical Centre, Adelaide). Patients were included if the echocardiogram demonstrated mild-to-moderate LVH (1.2–1.5 cm). Exclusion criteria included greater than mild severity aortic valve disease, dilated, restrictive, hypertrophic or infiltrative (eg amyloidosis, sarcoidosis) cardiomyopathy and malignant hypertension. Patients fulfilling the inclusion and exclusion criteria were then invited by letter to participate and only included if they contacted the study investigators with a positive response. This study was approved by the Institutional Ethics Committee inclusive of informed patient consent.

Screening for FD was conducted by measuring AGA activity with the fluorogenic substrate in 3 mm dried filter paper blood spots (DBS) from a finger-prick of blood essentially as described previously [6], with β-galactosidase measured as the control enzyme to confirm sample integrity. Lyso-Gb3 was quantified in plasma as detailed by Talbot et al. [7]. Next generation sequence analysis was performed using the 552 gene Illumina TruSight™ Inherited Disease Panel on the Illumina NextSeq® Sequencing system. Analysis of the resultant sequence data was restricted to *GLA*. Confirmatory testing of the detected variants was by Sanger sequence analysis.

## 3 Results

From a total of 4099 invited to participate in the study, only 511 (13%) accepted and due to the higher prevalence of LVH in males, 332 (65%) were male and 179 (32%) were female. The mean (range) age of women was 64 years (36–75) and men 63 years (18–75) (Table 1). In the male cohort, 2 patients returned enzyme activity below the reference interval of >2 nmol/h/mL; one aged 71 years at 0.8 nmol/h/mL, which was 19% of the mean of normal (patient 1) and the other aged 69 at 0.2 nmol/h/mL, which was 5% of the mean of normal (patient 2). Plasma lyso-Gb3 was normal in both patients. Sequencing of the *GLA* gene identified the p.Ala143Thr variant in patient 1 and p.Asn215Ser in patient 2 (Table 2). CMR findings for patient 1 included increased septal wall thickness (1.4 cm), normal LV mass (57 g/m^2^), subtle LGE in the basal inferolateral wall and normal T1 values except for a slight reduction noted in the mid-ventricular inferolateral wall. CMR for patient 2 was not performed due to a CMR incompatible permanent pacemaker.

**Table 1:** Baseline characteristics of LVH patient cohort.

Variables	Total cohort (*N* = 511)	Males (*N* = 332)	Females (*N* = 179)
Age (years)	64 ± 8.6 (65)	63 ± 8.68 (65)	64 ± 8.46 (66)
	[18–75]	[18–75]	[36–75]
Enzyme activity(nmol/h/ml)	4.37 ± 1.76 (4.1)	4.19 ± 1.49 (4.0)	4.69 ± 2.14 (4.3)
	[0.2–19.8]	[0.2–10.1]	[1.7–19.8]
IVS (cm)	1.28 ± 0.11 (1.2)	1.30 ± 0.11 (1.3)	1.26 ± 0.1 (1.3)
	[1.2–1.5]	[1.2–1.5]	[1.2–1.5]
PW (cm)	1.24 ± 0.08 (1.2)	1.25 ± 0.08 (1.2)	1.22 ± 0.05 (1.2)
	[1.2–1.5]	[1.2–1.5]	[1.2–1.5]
Wall thickness (cm)	1.26 ± 0.08 (1.3)	1.27 ± 0.08 (1.3)	1.24 ± 0.06 (1.2)
	[1.2–1.5]	[1.2–1.5]	[1.2–1.4]

mean ± SD, (median), [min-max] is shown. IVS, Intraventricular septum; PW, posterior wall thickness; Wall thickness, average (IVS + PW).

**Table 2:** Individual characteristics of the two male FD patients.

Patient	Age	Sex	Genotype	enzyme (nmol/h/ml)	lyso-Gb3 (nmol/L)	IVS (cm)	PW (cm)	GLS	Clinical symptoms
1	71	M	c.427G > A (p.Ala143Thr)	0.8	<5	1.5	1.4	−14	Mild concentric LVH; ECG: sinus rhythm, mildly prolonged PR interval; syncope; AV block; hearing loss
2	69	M	c.644A > G (p.Asn215Ser)	0.2	<5	1.2	1.2	−15	Mild concentric LVH; ECG: paced rhythm, normal PR interval; vertigo/dizziness

IVS, Intraventricular septum; PW, posterior wall thickness; GLS, left ventricular global longitudinal strain (reference range − 19% ± 3); LVH, left ventricular hypertrophy; AV, atrioventricular block.

In the female cohort, one 58 year old returned enzyme activity of 1.7 nmol/h/mL but was lost to further follow-up. Given the lack of sensitivity of AGA activity for females, the bottom 5% of enzyme activity results were reflexed for plasma lyso-Gb3 determination and *GLA* sequenced. This comprised a total of 10 females with enzyme activity in the range of 2 to 2.4 nmol/h/mL, but only 5 consented, all returning lyso-Gb3 within the reference range and no *GLA* variants identified.

## 4 Discussion

Testing for FD in males with enzyme determinations from DBS is a fast, cheap and simple approach, easily adaptable to mass population screening programs and does not involve venipuncture. Unlikely to miss male patients, as all should return activities below the reference range, confirmatory testing is recommended for a diagnosis of FD [8]. In this cohort of 332 males with unexplained LV wall thickening, there were 2 cases (0.6%) of reduced enzyme activity. Confirmatory biochemical testing with plasma lyso-Gb3 was unremarkable, and the only informative secondary testing was provided by molecular analysis of *GLA*. Genetic variants were detected in both patients and although p.Asn215Ser is well established as inducing a late onset cardiac variant of FD the significance of p.Ala143Thr, despite being a relatively common variant, is controversial with conflicting evidence of pathogenicity [9]. Initially believed to be pathogenic, a comprehensive analysis of the expression of this genetic variant then redefined it as benign. However, more recently p.Ala143Thr has been shown to be associated with FD cardiomyopathy with incomplete age- and gender- related penetrance [10].

A limitation of our study is the poor uptake rate with only 1 in 8 individuals responding to the invitation to participate. Comparisons between responders and non-responders showed no differences in age or gender suggesting that the included cohort is likely representative for the population targeted. Another significant limitation of our approach is that enzyme activity determinations for females are typically unreliable therefore we cannot rule out that heterozygotes were missed. Plasma lyso-Gb3 has demonstrated utility for identification of at least some heterozygotes with normal AGA activity [7] but the limited number of lyso-Gb3 confirmatory tests performed in this study was not informative. Next generation sequencing, which would be expected to detect at least 95% of FD variants (a proportion of intronic variants, promoter variants, large deletions or chromosome rearrangements may not be identified), did not reveal the presence of any known or potentially pathogenic sequence variants in the five female patients tested.

It is noteworthy that patients with significant hypertension (defined as using more than one anti-hypertensive) were excluded from this study in the belief that this pathology is causative for LVH. However, Terryn et al. [11] included patients with hypertension when testing for FD and from a total of 540 patients (362 males and 178 females) identified six patients with *GLA* variants, all of whom had hypertension. This is considerably higher than the two patients identified in our study from a similar sample size. This suggests that FD should remain in the differential for patients with hypertension. Furthermore, Monserrat et al. [12] reported 1% prevalence rate of FD in 508 patients with hypertrophic cardiomyopathy, likely higher than our study because patients with previously diagnosed cardiomyopathy with more severe LVH (maximal wall thickness > 1.5 cm) were included. A small study identified two patients from a cohort of 79 males with unexplained LGE on CMR, translating to 2.5% [5]. Another recent study of 266 patients with LVH identified five patients (2%) all with the intronic IVS4 + 919G > A genotype, known to be associated with late onset FD [13]. These five individuals were all of Chinese origin, a population where the IVS4 + 919G > A variant has a particularly high frequency [14]. On the other hand, another study by Schiffman et al. [15] screened a larger cohort of 2256 consecutive patients with common heart disease inclusive of all forms of cardiovascular implications and identified no FD patients. Taken together, these studies suggest that screening selected high-risk populations with specific abnormalities such as non-ischemic cardiomyopathy and positive, otherwise unexplained LGE on CMR improves the diagnostic yield for FD. Whether this also extends to screening low T1 values on CMR (as typically seen in early stage FD) in the absence of a family history and other clinical correlates and/or LGE positivity requires further study.

In conclusion, our study emphasizes the importance of considering FD as a differential, even in patients with mild-to-moderate LVH thought to have a low suspicion of the condition.

## Author contributions

Maria Fuller, Janice Fletcher and Joseph Selvanayagam designed the study. Rebecca Perry and Madiha Saiedi recruited patients and collected data. Maria Fuller, Rebecca Perry and Madiha Saiedi analysed the data and wrote the manuscript. All authors critically reviewed and contributed intellectually to the final version.

## Declaration of Competing Interest

None.
